# Preventable COVID-19 cases and deaths by alternative vaccination and non-pharmacological intervention policies in Brazil

**DOI:** 10.1590/1980-549720230054

**Published:** 2023-12-01

**Authors:** Samantha Rodrigues de Araújo, João Flávio de Freitas Almeida, Lásara Fabrícia Rodrigues, Elaine Leandro Machado

**Affiliations:** I Universidade Federal de Minas Gerais, Department of Industrial Engineering – Belo Horizonte (MG), Brazil.; II Universidade Federal de Minas Gerais, Faculty of Medicine – Belo Horizonte (MG), Brazil.

**Keywords:** Health planning, COVID-19, Vaccines, Program evaluation, Epidemiological model, Planejamento em saúde, COVID-19, Vacinas, Avaliação de programa, Modelo epidemiológico

## Abstract

**Objective:**

This work aimed to estimate the avoidable COVID-19 cases and deaths with the anticipation of vaccination, additional doses, and effective non-pharmacological interventions in Brazil.

**Methods:**

We developed a susceptible-exposed-infectious-recovered-susceptible model based on epidemiological indicators of morbidity and mortality derived from data obtained from the Health Information System of the Ministry of Health of Brazil. The number of cases and deaths was estimated for different scenarios of vaccination programs and non-pharmacological interventions in the states of Brazil (from March 8, 2020, to June 5, 2022).

**Results:**

The model-based estimate showed that 40 days of vaccination anticipation, additional vaccine doses, and a higher level the nonpharmacological interventions would reduce and delay the pandemic peak. The country would have 17,121,749 fewer COVID-19 cases and 391,647 avoidable deaths

**Conclusion:**

The results suggest that if 80% of the Brazilian population had been vaccinated by May 2021, 59.83% of deaths would have been avoided in Brazil.

## RESUMO

**Objetivo:**

Este trabalho visou estimar os casos e óbitos evitáveis de COVID-19 com a antecipação da vacinação, doses adicionais de vacinas e intervenções não farmacológicas eficazes no Brasil.

**Métodos:**

Propôs-se um modelo suscetível-exposto-infectado-recuperado-suscetível baseado em indicadores epidemiológicos de morbidade e mortalidade obtidos de Sistemas de Informação em Saúde do Ministério da Saúde do Brasil. O número de casos e mortes evitáveis foi estimado para diferentes cenários de programas de vacinação e intervenções não farmacológicas nos estados do Brasil (de 8 de março de 2020 a 5 de junho de 2022).

**Resultados:**

A estimativa baseada no modelo mostrou que 40 dias de antecipação da vacinação, doses adicionais de vacina e um nível mais alto de intervenções não farmacológicas reduziriam e retardariam o pico da pandemia. Haveria 17.121.749 casos a menos de COVID-19 e 391.647 mortes evitáveis no país.

**Conclusão:**

Os resultados sugerem que, se 80% da população brasileira tivesse sido vacinada até maio de 2021, haveria 59,83% de mortes evitadas no Brasil.

## INTRODUCTION

Since February 2020, the world has been ravaged by the COVID-2019 pandemic, with over 635 million confirmed cases and 6.61 million confirmed deaths worldwide^1-3^. In the lack of COVID-19 vaccines, governments issued non-pharmaceutical interventions (NPIs)^4-8^. NPIs are a set of different possible interventions, such as mask use, school closures, and lockdowns, that affect the degree of quarantine, social distancing, and mobility rates, reducing transmissibility. During the entire pandemic period, the NPIs prevented a global health catastrophe.

Uncertainty surrounding COVID-19 and its treatment challenged healthcare professionals and policymakers^5,6^. Countries’ healthcare systems faced beds and ventilators shortages^5,9^. At the political level, countries implemented social distancing measures and other NPIs, such as lockdowns and distancing regulations (restricting travel, mass gatherings, closure of workplaces/schools), mandatory use of masks, and hand hygiene with alcohol to slow the spread of the pandemic^4,5,9-14^. Despite their proven effectiveness in reducing virus transmission and deaths, oscillating strategies carried economic and humanitarian costs, ranging from unemployment to depression and anxiety^5,6^.

Countries such as Taiwan and South Korea extensively tested and isolated the infected, and European countries, such as Spain, France, the United Kingdom, Germany, and Italy, imposed restrictive lockdowns to avoid the virus’ spread^7^. On the other hand, Brazil, Sweden, and the United States adopted a less comprehensive approach reacting late to the epidemic and allowing infections to increase rapidly^5,7^, which might have resulted in an excess of cases and deaths^12,14^.

In December 2020, Regulatory Agencies approved COVID-19 vaccines, and vaccination started in many countries^2,3,15^. Russia started its vaccination programs on December 5^th^, 2020, and the United Kingdom, the United States, and the United Arab Emirates on December 14^th^, 2020. Latin American countries such as Chile, Argentina, and Mexico started vaccination on December 24^th^, 2020. On the other hand, in the American continent, Brazil was one of the nations that postponed the beginning of the vaccination program, starting only on January 18^th^, 2021^1,15,16^.

Despite having one of the world’s most successful immunization programs through the National Immunization Program (PNI) of the Ministry of Health, the vaccination strategy and the campaign against COVID-19 in Brazil were implemented slowly and not timely, in contrast to previous successful vaccination campaigns, such as the 2009 H1N1 influenza pandemic, in which 89 million doses of influenza vaccine were administered in three months^17^. Aspects such as the lack of coordination and support for scientific research from the federal government and the oscillating and relaxed application of NPIs^18,19^ resulted in 655,000 deaths from COVID-19 by June 2022^1,16^. To improve future NPI policies and vaccination, we need to estimate the number of SARS-CoV-2 virus cases and deaths that could have been avoided if the NPIs strategy and the COVID-19 vaccination had been effectively implemented. Therefore, we simulated the dynamics of the COVID-19 pandemic in Brazil, using the susceptible-exposed-infected-recovered-susceptible (SEIRS) model for different vaccination and NPIs strategies.

The SEIR modelling approach was used to estimate the progression of the COVID-19 pandemic and to discuss the control strategies when implementing social distancing, periods of school closures, and human mobility patterns measures^20-30^. The studies proposed measures to reduce the height of the peak to allow more time for health systems to expand and respond. The present study contributes to evaluate the impact of NPIs on the progress of the pandemic. This work’s scientific contribution reveals the potential of an earlier vaccination program to prevent cases and deaths.

This work aims to estimate the number of preventable cases and deaths from COVID-19 upon vaccination programs and non-pharmacological interventions in Brazil.

## METHODS

### Data sources and measurements

This work uses daily historical data from each state for the number of cases (infections), deaths (absolute number of deaths), and number of vaccinated individuals available at https://github.com/wcota/covid19br ^31^ which aggregates data from at least two main sources^2^: the Ministry of Health^1^ and Brasil.IO^32^. Previous works use the same data source in their analysis (see, for example, Araújo et al.^33^, Badr et al.^34^, Cassão et al.^35^, Aragão et al.^36^, and Almeida et al.^37^).

**Figura 1.**
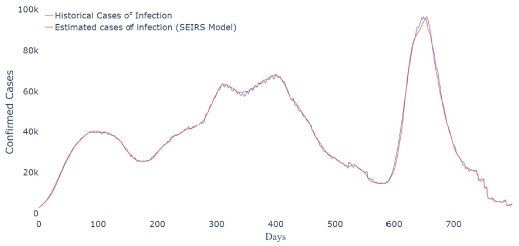
Estimated cases of infections in Brazil using the susceptible-exposed-infected-recovered-susceptible model.

The study used data from March 2020 to June 2022 from the 26 Brazilian states and the Federal District (DF) to develop the SEIRS model. First, we estimated the parameters of the SEIRS model for each state. Then, we used this parameter to estimate the cases of infection in Brazil (Figure 1).

The COVID-19 Pandemic Parliamentary Commission of Inquiry (CPI COVID-19)^(16)^ addressed the delay in the Brazilian vaccination program, stating that Brazil could have started the vaccination program in the first half of December 2020, like most Latin American countries (see page 239 of the report). Therefore, as indicated in the CPI report, we anticipated the vaccination schedule in Brazil to December 8^th^, 2020 (a possible date).

We estimated the number of cases and deaths for three possible scenarios of vaccination programs and NPI strategies in 819 days of the pandemic: from March 8^th^, 2020, to June 5^th^, 2022.

Scenario 1 [Vaccination anticipation]: Vaccination from the first possible day in Brazil (2020, December 8^th^) without adding doses.

Scenario 2 [Vaccination anticipation and additional doses of vaccines]: This scenario assumes the anticipation of the vaccination program to December 8^th^, 2020, and the availability of vaccine doses to vaccinate 80% of the Brazilian population (delivered in early December 2020 until May 31, 2021; see Annexes in the Supplementary Material).

Scenario 3 [Vaccination anticipation, additional doses of vaccines, and effective NPIs]: Besides anticipating the vaccination program to 2020 December 8^th^ and the additional vaccine doses, we propose Scenario 3 by changing the NPIs to a 34.22% higher effectiveness. We assume that a higher NPI effectiveness is a 34.22% lower number of deaths obtained by the city of Belo Horizonte compared to other Brazilian capitals with more than one million inhabitants^38^.

### Susceptible-exposed-infected-recovered-susceptible model: capturing the effects of vaccination and non-pharmaceutical interventions

**Figura 2.**
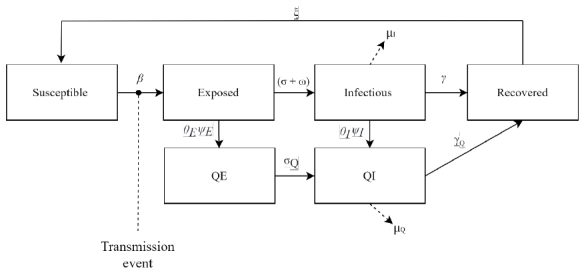
Susceptible-exposed-infected-recovered-susceptible diagram.

The epidemiological SEIRS model (Figure 2) predicts infectious disease dynamics by compartmentalizing the population. The model is governed by a system of ordinary differential equations (1–7)^39^, where S, E, I, R, and F are the amount of susceptible, exposed, infectious, recovered, and deceased individuals, respectively; N is the total number of individuals in the populations; *Q_E_* are the exposed quarantined individuals ; *Q_I_* are the infectious quarantined individuals; *β* is the rate of transmission (exposure); *β_Q_* is the rate of transmissibility of quarantined individuals; *σ* is the rate of infection (upon exposure); *σ_Q_* is the rate of progression to infectiousness for quarantined individuals (inverse of the latent period); *γ* is the rate of recovery (upon infection); *γ_Q_* is the rate of recovery for quarantined individuals (inverse of the infectious period); *γ_I_* is the rate of recovery for infected individuals; *ξ* is the rate of re-susceptibility (inverse of temporary immunity period; 0 if permanent immunity period) (upon recovery); μ_I_ is the rate of infection-related death; μ_Q_ is the rate of death for quarantined individuals; *ω* is the rate of infection by new variants; and *q* is the weight of intensity of global interactions for individuals in quarantine.

The proposed model distinguishes the individuals into two groups throughout the pandemic: in quarantine, i.e., a group created from the tested population; and out of quarantine. Exposed and infectious individuals are tested at rates *θ_E_* and *θ_I_*, that test positively for infection with probabilities *ψ_E_* and *ψ_I_*, respectively (the false positive rate is assumed to be zero). A positive test result moves an individual into the appropriate quarantine state and the individuals remain in isolation until their designated isolation time has been reached or until they recover. In addition to addressing quarantine, the model introduces elements such as social distancing and the transmissibility rate. There are groups at higher risk of death, such as the elderly and individuals with comorbidities and social vulnerabilities^(40,41)^. We adopted an average rate of deaths per infected population.


(1) $$\overset{\cdot }{S}=\frac{-\beta SI}{N}-q\frac{\beta_{Q}SQ_{I}}{N}$$

(2) $$\overset{\cdot }{E}=\frac{-\beta SI}{N}+q\frac{\beta_{Q}SQ_{I}}{N}-\sigma E-\theta_{E}\psi_{E}E$$

(3) $$\overset{\cdot }{I}=(\sigma +\omega )E-\gamma I-\mu_{I}I-\theta_{I}\psi_{I}$$

(4) $${\overset{\cdot }{Q}}_{E}=\theta_{E}\psi_{E}E-\sigma_{Q}Q_{E}$$

(5) $${\overset{\cdot }{Q}}_{I}=\theta_{I}\psi_{I}I-\sigma_{Q}Q_{E}-\gamma_{Q}Q_{I}-\mu_{Q}Q_{I}$$

(6) $$\overset{\cdot }{R}=\gamma I+\gamma_{Q}Q_{I}$$

(7) $$\overset{\cdot }{F}=\mu_{I}I+\mu_{Q}Q_{I}$$

(8) $$N=S+E+I+Q_{E}+Q_{I}+R$$

We propose an extended version of the SEIRS model by incorporating the virus transmission rate varying in time (β), the vaccination program, and the effect of NPIs in the number of cases and deaths on the curve of COVID-19 for all Brazilian states. Hence, the curve of the cases estimated by the SEIRS model is modeled according to the five parameters that vary in time: β, ω, *E_dist_*, *E_vac_*, and *P_vac_*. In this work, we modeled these parameters varying over time to address the different stages of the pandemic. The parameters β, ω, *E_dist_*, and *E_vac_* were adjusted using historical data of reported infections referring to two and a half years of the pandemic in Brazil. On the other hand, the *P_vac_* is a parameter obtained directly from the data collected^31^.

The rate of transmission (β) is a key parameter in determining how fast COVID-19 can spread through the population during the early stages of the disease. Its estimation is inherently challenging since the reported cases are likely to be a smaller fraction of real cases^42^ and the real number of cases and their changes over time is unknown.

We also incorporate the effect of NPIs into the SEIRS model by introducing *P_E_* as the percentage of the exposed population in Equation 9. In this case, the effect of the NPIs is represented by ,*E_dist_* the effectiveness of social distancing.


(9) $$P_{E}=1-E_{dist}$$

Moreover, the effect of the vaccine is included in Equation 10, where *P*_s_ is the percentage of the susceptible population; *P*_vac_ is the percentage of the vaccinated population^2,3^, and *E*_vac_ is the vaccine effectiveness. We also assume that vaccinated and unvaccinated individuals are susceptible to the virus with different probabilities.


(10) $$P_{S}=P_{E}*(1-P_{vac}*E_{vac})$$

The estimated number of deaths (*M*) is presented in Equation 11, in which *λ* is the death rate of vaccinated individuals^2,3,43^, ρ is the death rate of unvaccinated individuals^2,3,43^, and *C_P_* is the number of predicted cases.


(11) $$M=[\rho *C_{P}*(1-P_{vac})]+[\lambda *C_{P}*P_{vac}]$$

Due to the lack of detailed data, we assume four simplifying assumptions. First, the population is homogeneous, i.e., all individuals have the same infection rate and parameters. Second, all individuals are equally likely to interact with all other individuals. Third, this model assumes that individuals are tested randomly at exponentially distributed intervals corresponding to mean testing rates. Finally, the model assumes a population uniformly dispersed in a geographical area, despite the fact that urban centers with a greater concentration of population can present a higher probability of infection than areas with a smaller number of people.

The model was implemented in Python software (Python Language Reference, version 3.10.4). The code is available in Supplementary Materials.

The criterion used to evaluate the predictive performance of the SEIRS model was the mean absolute percentage error (MAPE). MAPE was calculated using Equation 12, in which *P_t_* is the predicted value at time *t*, *Z_t_* is the observed value at time *t* and *T* is the number of predictions.


(12) $$MAPE=\frac{1}{T}\sum_{t=1}^{T}\left|\frac{\left(P_{t}-Z_{t}\right)}{Z_{t}}\right|$$

## RESULTS

Brazil had a cumulative infection quantity of 30,733,955 cases and 654,572 deaths by June 2022^1-3^, with 2.57% deaths per infected person (lethality rate)^43^. The model presented a MAPE of 5.12% for all of Brazil, suggesting good accuracy in predicting epidemic diseases based on the results of Zhang et al.^44^ We present the results for the three scenarios using a 30-day moving average to plot the curve of the cases for better curve smoothing.

**Figura 3.**
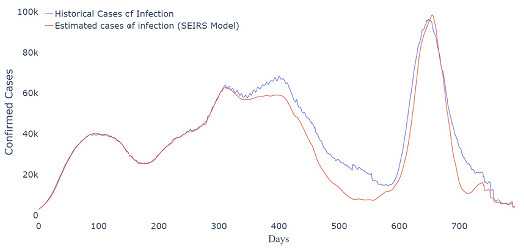
Estimated infection curve of Scenario 1.

**Figura 4.**
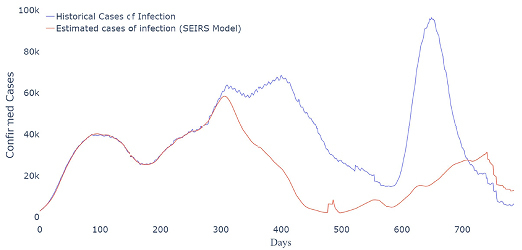
Estimated infection curve of Scenario 2.

**Figura 5.**
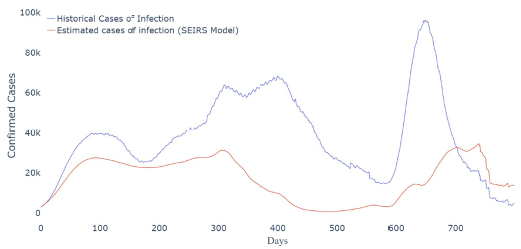
Estimated infection curve of Scenario 3.

- Scenario 1 [Vaccination anticipation]: The model estimates that 3,517,329 cases and 86,639 deaths could have been avoided (Figure 3).
- Scenario 2 [Vaccination anticipation and additional doses of vaccines]: As a result, the model estimates that 11,697,890 cases and 266,953 deaths would be avoided during the analyzed period (Figure 4).
- Scenario 3 [Vaccination anticipation, additional doses, and effective NPIs]: The result of this scenario estimates the prevention of 17,121,749 cases and 391,647 deaths. The estimated infection curve to Scenario 3 is presented in Figure 5.

Table 1 summarizes the SEIRS model estimates for the three scenarios considering all Brazilian states. In this sense, our simulations show that control measures aimed at timely vaccination (early), availability of additional doses, and more robust NPIs could have significantly prevented the number of cases and deaths from COVID-19 in Brazil.

**Table 1.** Simulated scenarios

Items	Vaccination start date	Strategies	Number of estimated cases	Number of estimated deaths	Infection avoided	Deaths avoided
Base	01/18/2021	-	30,733,955*	654,572*	0	0
Scenario 1	12/08/2020	Vaccination anticipation	27,216,626	567,933	3,517,329	86,639
Scenario 2	12/08/2020	Vaccination anticipation Additional doses of vaccines	19,036,065	387,619	11,697,890	266,953
Scenario 3	12/08/2020	Vaccination anticipation Additional doses Effective NPIs	13,612,206	262,925	17,121,749	391,647

* Historical data from official records^1-3^.

## DISCUSSION

COVID-19, a contact-transmissible infectious disease, spreads through a population via direct contact between individuals^9,20^. Control measures are applied to avoid cases and deaths from the disease. This work aims to estimate the number of preventable cases and deaths from COVID-19 upon alternative control measures of vaccination and NPIs in Brazil. Therefore, we modelled the real curve of cases and deaths and evaluated the effect of different vaccination strategies and NPIs measures. To this end, we used the SEIRS epidemiological model by estimating pandemic parameters. The simulation shows that intense control measures of NPIs and anticipation of vaccination would have reduced cumulative infections by the end of 2020 while also delaying the peak of the disease.

Scenario 1 shows the avoidable number of cases and deaths if the vaccination program had been implemented with no additional doses of vaccine, i.e., if the country had implemented all the previous strategies in addition to anticipating the start of vaccination. In this scenario, we observe that 86,639 (13.23%) deaths could have been avoided, showing the importance of timely time vaccination strategies.

Comparing the strategy adopted in Brazil with the results obtained in Scenario 2, 38.06% of cases and 40.78% of deaths could be avoided by anticipating vaccination and purchasing additional vaccine doses. Our projections show that the deaths could be substantially decreased if the population’s vaccination coverage was higher until May 2021.

Similar works by Ferreira et al.^45^, Santos et al.^46^, Santos et al.^47^, and Orellana et al.^48^ demonstrate the direct impact of COVID-19 vaccination in reducing the number of deaths. Ferreira et al.^45^ and Orellana et al.^48^ also evaluate changes in hospitalization patterns, and Santos et al.^46^ and Santos et al.^47^ analyze the reduction in severe cases, both associated with vaccination.

The vaccination against COVID-19 in Brazil started almost one year after the beginning of the pandemic. During this period, control of the infection was carried out only by the NPIs. Brazil reacted ineffectively to the pandemic compared to most countries through oscillating NPIs^7,16^. Strategies considered “flexible or relaxed” did not prevent increasing cases and deaths^5^. In this sense, we observed that if the municipalities had adopted effective NPIs combined with a timely vaccination program and additional doses of vaccines, 17,121,749 cases of COVID-19 would have been avoided and 391,647 deaths would have been avoided, i.e., the country would have avoided 55.70% of cases and 59.83% of deaths by adopting Scenario 3 compared to the baseline one. Scenario 3 also highlights the importance of implementing NPI strategies. Comparing its results with Scenario 2, 17.64% of cases and 19.04% of deaths could have been avoided by implementing these strategies.

In this context, Genari et al.^49^ address the safety of school activities considering the implementation of NPIs and vaccination, associating the adoption of effective NPI protocols with a reduction in the number of cases. On the other hand, Werneck et al.^19^ and Silva et al.^50^ estimate the number of cases and deaths that could be avoided in Brazil if only NPIs were used to control the pandemic.

In terms of the research method, the works that addressed this issue adopted methodologies such as the SEIR^(49,51)^, statistical methods^(45-47)^, exploratory analysis^(19,50)^, and ecological study^(48)^. Genari et al.^(49)^ use the SEIR method without assuming that individuals become susceptible again after recovery. On the other hand, based on Silva et al.^(51)^, we assume that vaccinated individuals can still become infected and in some cases even die.

Although the effects of interventions may vary among the countries, our approach is flexible enough to describe different pandemics or epidemics and to evaluate alternative scenarios in these situations. The proposed model provides better estimates of disease progression and highlights the usefulness of appropriate population vaccination programs and NPIs. Therefore, health planners can use it to manage future pandemics and epidemics.

We also assumed that 80% of vaccination coverage would be achieved by May 31^st^, 2021. However, the Brazilian health system, including research and healthcare infrastructure, has been underfunded in recent years^(52)^, which could significantly compromise the achievement of vaccination coverage within this period.

Our study has some limitations. Our model addresses multiple doses by assuming that all vaccinated individuals have taken two doses of vaccine, even as we know that not all vaccinated individuals have taken both doses and we do not focus on the necessity of each vaccine brand; however, other studies^(45,51)^ explicitly analyze the use of multiple doses. We also adopt as vaccine effectiveness the adjusted value for this parameter obtained using the collected data, without directly considering the number of vaccines of each brand used and the timely availability of each vaccine.

Another limitation of our work is the official data used to estimate the parameters of our model. Since the number of infections is not available, we assumed that the number of cases equals the number of infections. However, we recognize that this may underestimate the actual number, as underreporting and delays in the reporting are known and testing rates vary by region. For example, Paes et al.^(53)^ proposed a methodology to calculate the number of deaths from COVID-19, which shows a 37.4% increase compared to official records for Paraíba in Brazil. In this sense, our simulations represent a conservative scenario for the COVID-19 pandemic in Brazil, suggesting that the impact of NPIs and vaccination strategies would be higher than the results presented in this paper. Therefore, we recommend conducting a sensitivity analysis to assess the reliability of the results and provide insight into the robustness of the model.

Due to a lack of microdata, different infection rates were not applied to individuals based on health profile, age, or geographic location. Future work is required in this direction. Another challenging future research direction is to incorporate the changes in COVID-19 vaccine effectiveness over time into the modeling. Finally, we suggest future research on integrating the proposed SEIRS model into logistics vaccination networks for vaccine demand estimation and increasing its effectiveness in combating epidemics and pandemics.

## SUPPLEMENTARY MATERIALS:

Code and data: https://zenodo.org/record/7805587#.ZC7D33bMKUk.
